# Antifungal Potential of Nanostructured Crystalline Copper and Its Oxide Forms

**DOI:** 10.3390/nano10051003

**Published:** 2020-05-24

**Authors:** Auriane Fifame Oussou-Azo, Tomoki Nakama, Masayuki Nakamura, Taiki Futagami, Mun’delanji Catherine M. Vestergaard

**Affiliations:** 1 United Graduate School of Agricultural Sciences, Kagoshima University, Kagoshima 890-0065, Japan; oauriane1@gmail.com (A.F.O.-A.); masa@agri.kagoshima-u.ac.jp (M.N.); futagami@chem.agri.kagoshima-u.ac.jp (T.F.); 2 Faculty of Agriculture, Kagoshima University, 1-21-24 Korimoto, Kagoshima 890-0065, Japan; nt11uver@gmail.com

**Keywords:** antifungal, cell viability, nanostructured copper, copper nanoparticles, copper oxides, *Colletotrichum gloeosporioides*

## Abstract

Copper has been used as an antimicrobial agent for over a century and is now being added to commercial fungicides. Nanomaterials have attracted much attention due to the special properties they have over their bulk form. We studied nanostructured copper (Cu-NPs), investigating the potential for improved antifungal properties derived from its special properties and studied any effect that the oxidation of copper (CuO-NPs) may have. We conducted this research against *Colletotrichum gloeoesporioides*, a devastating pathogen to plants/crops worldwide. Research on the effects of copper on this fungus are limited. Our studies showed that nanoforms of copper had significant antifungal activities, with Cu-NPs offering the most sustainable efficacy and was more effective than its oxidative form (CuO-NPs). Scanning Electron Microscopy (SEM) images of the treated pathogen show that the hyphae had a swollen appearance, lost their filamentous structure, and the mycelia had a powder-like structure, indicating the probable destruction of the hyphal tubular cell wall. X-ray Difractogram (XRD) outputs showed substantial changes in the physical characteristics of the Cu-NPs after interaction with the fungus. This is the first report to demonstrate chemo-physical changes in the metal compounds, opening new insights for further studies on the mechanism of copper’s antifungal properties.

## 1. Introduction

For centuries, copper metal complexes have attracted enormous attention due to their biological and pharmacological properties, which provide them with diverse antimicrobial activities [1]. However, despite the fact that the healing properties of copper have been known for centuries, it has only recently that the metal was registered by the U.S. Environmental Protection Agency as the first solid antimicrobial material [2], and considerable research is ongoing to establish copper’s antimicrobial spectrum and mechanisms of action. Copper has been shown to effectively eradicate microorganisms and its compounds are used in the biomedical field [3,4,5], in air and water distribution systems to control healthcare-acquired infections [6,7,8,9], as biocides in agriculture and in marine antifouling paints [10], and in the textile industry with potential biocidal activities to fabrics [11,12].

Recently, nano-sized metal particles have caught the attention of researchers because of their unique physical and chemical properties, which are dissimilar from the bulk metal. The nanoparticle (NP) forms have been reported to enhance their antimicrobial abilities [13,14]. The enhanced antimicrobial ability is explained by the high surface area to volume ratio of the NPs, which gives them a larger active surface for interaction. Smaller NPs have been reported to breach cell membranes, thus disrupting intracellular process [15,16]. Indeed, Yadollahi et al. [17] reported that scaling down to NP sizes enhanced drug aqueous solubility and bioavailability by increasing the drug surface area that comes into contact with the biological media. Thus, metal NPs such as silver [18,19,20,21,22], copper [6,12,23,24,25,26], and gold [27,28] have been widely tested for their biological properties. During the past two decades, their applications have been directed toward developing therapeutic products targeting wound healing, cancer diagnosis and treatment, drug delivery, and control of microorganisms. Most of the research has focused on the antibacterial effect of Ag-NPs, and only a few on the effect of Cu-NPs. Indeed, relatively very little research has been conducted on the antifungal activities of copper and its nanoforms.

Fungal pathogens are widespread worldwide and cause many important diseases across a wide range of plant hosts. Indeed, filamentous fungi of the genus *Colletotrichum* are known to be the most destructive plant pathogens worldwide and are the responsible agent of anthracnose disease, one of the most economically important diseases that affect a wide range of crops including cereals, legumes, vegetables, and fruit crops [29,30,31,32,33,34]. Effective measures to curb *Colletotrichum* infections usually involve a combination of cultural, biological, and chemical controls [35,36,37,38]. These methods are laborious, expensive and some are environmentally unfriendly. Sanders et al. [39] previously reported that copper-based fungicides, when applied as pre-treatments, delayed the onset of anthracnose symptoms in mango and avocado plants in South Africa. Oziengbe and Osazee [40] successfully observed a reduction of *Colletotrichum gloeosporioides* (*C*. *gloeosp*.) growth and conidia germination when treated with copper sulfate. Meanwhile, nanoforms of copper have been reported to exert biocidal effects on different classes of fungi [41,42,43,44,45,46]. However, there are only a couple of reports on the influence of Cu-NPs on *C*. *gloeosp*. [36,47]. In addition, oxidative compounds of copper, copper oxide (CuO), have gained tremendous attention recently because it is the simplest member of copper compounds and possesses a range of potentially useful physical properties [25]. However, there are hardly any research reports on the possible antifungal activity of CuO and its nanoforms (CuO-NPs).

Thus, the present study investigated the effect of Cu-NPs, CuO-NPs, and CuO on anthracnose causal agent *C*. *gloeosp.* We studied the effect of the copper forms on mycelia growth, cell viability, and the pathogen’s microstructure. Furthermore, we studied the chemo-physical features of the copper forms to shed light into the possible mechanism of action. 

## 2. Materials and Methods

Various parameters including pH, degree and rate of aeration, amount of both fungus (spore density) and copper forms, types of copper used for treatment, and temperature effect of the interaction between the copper forms and the fungus. In this work, we looked at spore density, types of copper treatments, and the concentration of the coppers. We selected a temperature of 25 °C and pH of 7.4 and a naturally-aerated environment because these are close to the physiological and environmental conditions, making the results more applicable to real-life situations

### 2.1. Materials

*C*. *gloeosp*. was a gift from the Plant Pathology Laboratory, Faculty of Agriculture, Kagoshima University, Kagoshima, Japan. The gene sequences of the *C. gloeosp*. strain correspond to strain QPg961, GenBank accession EU200455. Copper compounds (Cu-NPs (Ca. No. 774111, 40–60 nm particles size), CuO-NPs (Ca. No. 544868, <50 nm particles size) and CuO (Ca. No. 203130)), potato dextrose broth (PDB), agar, and Alamar Blue Cell Viability Assay (AB) reagent were purchased from Sigma-Aldrich Co. Inc., Tokyo, Japan. Vegetable juice (V8 juice from Campbell) was purchased from a local supplier. All metal materials were used without further purification. Deionized water (Milli-Q water), resistivity 18.2 MΩ.cm was obtained from a Millipore Synergy UV Water Purification System (Millipore, Bedford, MA, USA). 

### 2.2. Preparation of Copper and Copper Compounds

Copper compounds were prepared in solution. The appropriate amounts of copper compounds were dissolved in Milli-Q water to reach finals concentrations of 50, 100, 200, 500, and 1000 mg/mL in media. The solutions were sonicated for 30 min at room temperature (RT) and used the same day.

### 2.3. Hyphal Growth Measurement

The antifungal activities of copper were tested against *C*. *gloeosp*. using the supplemented agar method. Molten Potato Dextrose Agar (PDA) was supplemented with each copper form solution at 50, 100, 200, 500, and 1000 mg/mL. A 4 mm diameter agar block was cut from the advancing edge of an actively growing fungal culture on PDA. Each agar block was placed in the center of an agar plate, the face with the pathogen down [19]. Plates were incubated at 25 °C for seven days, and used for the measurements of hyphal growth. A carefully calibrated in-house ruler was used to obtain the orthogonal diameter measurements of hyphal growth in mm. These were recorded for each fungal colony and the mean was used to calculate the percent growth. Each treatment was conducted in triplicate and the mean diameters were used to calculate the results.

### 2.4. Cell Viability Assays

Cell viability was measured using the AB assay, according to the manufacturer’s prescribed protocol (Sigma-Aldrich Co. Inc., Tokyo, Japan). Revealed to be safe for users, cells, and the environment, AB is a redox dye widely used in biomedical research to measure the in vitro viability, proliferation, and cytotoxicity of cells. The AB assay detection mechanism is subtended by the principle that the metabolic activity of living cells produces a reduced environment because oxygen is consumed. This leads to a decrease in the amount of the oxidized form (blue) of the dye, leading to an increase in a fluorescent intermediate (red) form. Reports on its use to evaluate its sensitivity to different fungi suggest that an optimization procedure to suit the target chemicals and specific pathogen is needed [36,48]. In our study, we undertook this procedure (see Section 2.4.1).

#### 2.4.1. Optimization of Culture Conditions

The choice of a suitable medium, optimal incubation period, and appropriate conidia concentration are very important for the AB assay. In our study, we used two types of media: PDA, because it is appropriate for the target fungus growth; and 10% V8 juice in Milli Q water (*v/v*) because this medium has been reported to support the best growth of *C. gloeosp*. for the purpose of spectrophotometric quantification [36]. Media were buffered with 0.165 M 3-morpholinopropane-1-sulfonic acid (MOPS acid, Sigma-Aldrich Co. Inc., Tokyo Japan) at 25 °C and the pH adjusted to 7.4 using sodium hydroxide. Media were autoclaved at 121 °C for 20 min and used on the same day.

Fungal isolates were routinely cultured using the two media following standard culture protocols. Stock suspension of the spores was prepared from a seven day-old culture by flooding a Petri dish with 10 mL of appropriate broth. After scraping the surface of the culture plates, the plates were vortexed to allow conidia to go into the suspension. The suspensions were strained using Mira cloth (Sigma-Aldrich, Tokyo, Japan) to remove the agar and mycelium. Conidia suspension concentration was determined using Countess (Automated Cell Counter, Thermo Fisher Scientific) and adjusted to 10^5^ spores/mL. Working suspensions were prepared using serial dilutions (10^1^ to 10^5^ spores/mL) and used the same day. For each concentration, a volume of 100 μL of each suspension was added to clear 96-well microplates. Ten μL of *AB* reagent was added to each well and the microplates were covered and placed in clean sealable plastic containers. Subsequently, the sets were incubated at 25 °C in the dark. Each test plate included a negative control (only medium without cells) to determine the background signal; and a positive control of 100% reduced the *AB* reagent without cells. All analyses were conducted in triplicate. Fluorescence readings were taken after 6, 12, 18, and 24 h, setting the excitation wavelength at 560 nm and emission wavelength at 590 nm using an Infinite M200 microplate reader (TECAN, Mannedoff, Switzerland). Percent reductions of *AB* (see equation in Supplementary Materials) were calculated and used to plot the calibration curves and determine the effective linear range.

#### 2.4.2. The Effect of Copper Nanoparticles and Oxide Forms on Cell Viability

We examined the viability of *C. gloeosp*. cells in the presence of copper nanoparticles and oxide forms (Cu-NPs, CuO-NPs, and CuO) using the *AB* assay. Copper suspensions were plated with appropriate conidia suspension using optimized medium and spore density to reach final concentrations of 200 and 500 mg/mL. These concentrations were selected based on the results obtained from hyphal growth measurements. Test wells containing copper solutions and fungal spores were plated in triplicate. Each test plate included a positive control (containing medium and conidia suspension), negative control (only medium), and chemical control (copper suspensions and medium) wells. Ten μL of *AB* reagent was added to all wells, the microplates covered, and placed in sealable containers. The containers were gently rotated horizontally and incubated at the optimized incubation period (24 h). Visual inspection and fluorescence measurements were taken (λ_cm_ 560 nm and λ_ex_ 590 nm); and descriptive statistics calculated. Thereafter, the percent difference in the reduction of *AB* dye was calculated following the equation provided by manufacturer (see Supplementary Materials).

### 2.5. X-ray Diffractogram (XRD) Characterization

All copper forms were characterized before and after interaction with the pathogen using XRD. The x-ray diffractograms were obtained with a PANalytical X’PERT PRO MPD diffractometer (Malvern Panalytical, Malvern, UK) operating at 45 kV and 40 mA with Cu-Kα radiation (λ = 1.54060 Å) at a scan speed of 0.01 from the 2θ 20° to 80° range. Pathogens were cultured in PDB in the presence of the different copper forms at the final concentration of 500 mg/mL. After seven days of incubation, mycelia were removed from the liquid media. Then, the liquid media containing the copper materials were poured through filter paper, easily trapping the coppers on the filter papers. The coppers were re-dispersed in distilled water and recollected after centrifugation at 3000 rpm for 10 min. Afterward, they were dried on a slide glass and analyzed. Diffractograms were obtained and analyzed.

### 2.6. Scanning Electron Microscopy (SEM)

Fungus micrographs were obtained using a scanning electron microscope (SEM; FEI Quanta 400 (Thermo Fisher Scientific, Tokyo, Japan)) operating in low vacuum with water vapor and an accelerating voltage of 10 kV. Before the SEM analysis, an approximately 4 mm diameter agar block of *C. gloeosp*. was cultured in liquid medium supplemented with different copper forms at a final concentration of 500 mg/mL. After seven days of growth, mycelia were collected using filter paper and cut into blocks of about 5 mm × 5 mm. Each specimen was carefully placed on the microscope holder with carbon tape.

### 2.7. Statistical Analyses

Data were analyzed using IBM SPSS Statistics Version 23 packages for Mac. For the effects of various forms of copper and their concentrations on pathogen hyphal growth and cell viability, all experiments were conducted in triplicate and mean values were considered. ANOVA analyses were used assuming a *p-*value of 0.05.

## 3. Results

### 3.1. Dose-Dependent Effects of Copper, and Its Oxidative Forms on Fungal Hyphal Growth

**Figure 1:**
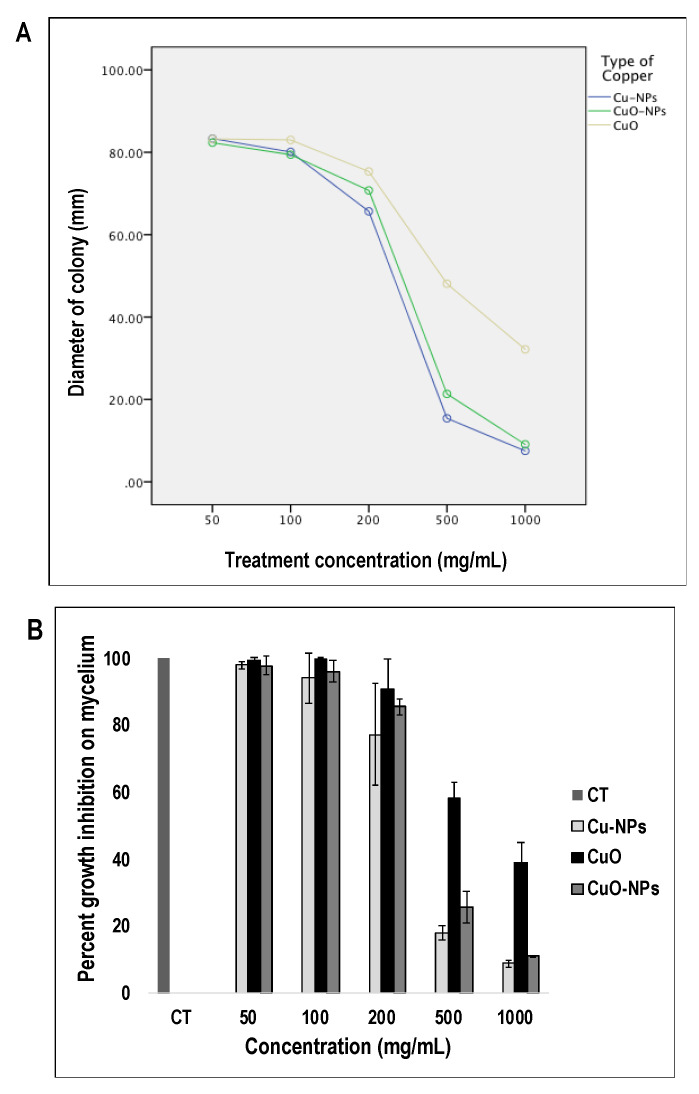
Effects of copper forms (Cu-NPs, CuO-NPs, CuO) at various concentrations of 0 (CT), 50, 100, 200, 500, and 1000 mg/mL on *C. gloeosp.* hyphal diameter growth. *C. gloeosp.* was grown in PDA for seven days at 25 °C. (**A**) Pathogen colony diameter in presence of tested copper forms; (**B**) Percent growth inhibition of pathogen hyphae by copper treatments at different concentrations.

We tested the effects of various concentrations of copper forms on *C. gloeosp*., as indicated by changes in hyphal growth in amended PDA. All copper forms caused a marked decrease in the radial growth of the pathogenic fungus at concentrations of 500 mg/mL and above (Figure 1A). Analysis of variance (ANOVA) revealed that copper forms, their respective concentrations, and the interaction between both factors had significant effects on the pathogen hyphal growth, *p* < 0.05 (Table 1). Cu-NPs and CuO-NPs caused the largest effect with 81.9 and 74.2 growth percentage inhibition at 500 mg/mL, respectively; and 91.2% and 89% at 1000 mg/mL, respectively (Figure 1B). Although Cu-NPs had a higher inhibiting effect than CuO-NPs, this was not statistically different (*p* < 0.05). The bulk form of CuO had significantly (*p* < 0.05) less inhibitory effect than the nanostructured and decreased the pathogen growth by 41.8% and 61.6% at 500 mg/mL and 1000 mg/mL, respectively. Between 50 and 200 mg/mL, the hyphae growth was only slightly inhibited by all copper forms.

### 3.2. Effects of Copper NPs and Oxide Forms on Pathogen Cell Viability

**Figure 2:**
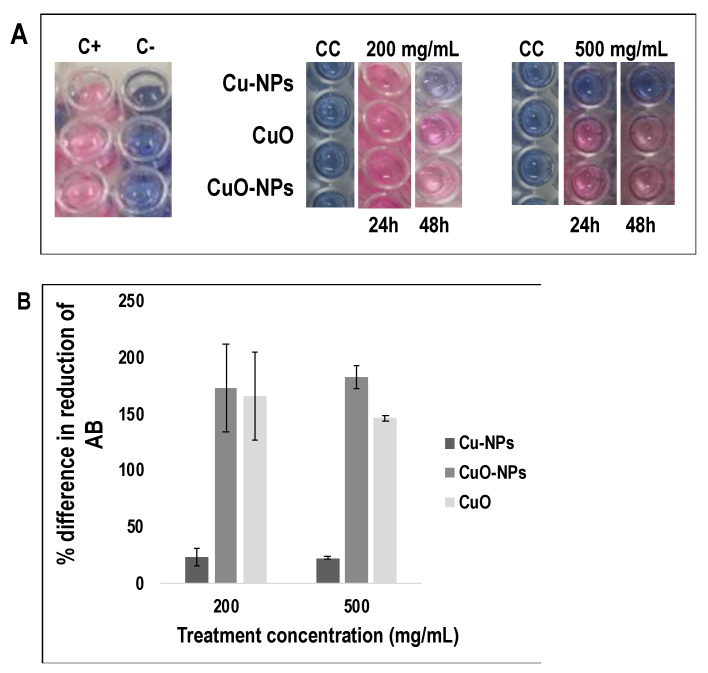
Effects of copper forms on the cell viability of *C. gloeosp* measured using the Alamar Blue Assay in conjunction with a fluorescence spectrophotometer. Method optimized from the user’s manual (Sigma-Aldrich Co. Inc., Tokyo, Japan). (**A**) Visual color change of the Alamar Blue dye incubated with pathogen spores and various forms of copper (Cu-NPs, CuO-NPs, and CuO) at different concentrations; C^+^ is the sample control (medium + spores); C^−^ is a reagent control (only medium); and CC is the treatment control (treatment + medium). (**B**) Percent difference in the reduction of Alamar Blue dye between the treated and control spores. The data shown are the mean of three replicates and error bars represent standard deviation.

The AB assay was used to analyze the effect of the coppers on the viability of *C. gloeosp*. cells. First, we optimized the AB assay conditions considering parameters such as medium type, incubation period, and spore density; thus ultimately selecting the best combination for the cell viability test. The best combination was a spore density of 10^5^ spores/mL, PDA media, and an incubation period of 24 h (please refer to the Supplementary Materials for details). We then conducted negative and positive controls to confirm that neither the medium nor copper forms provided a reducing environment to *AB* in the absence of viable spores (Figure 2A). 

In the presence of copper forms, only Cu-NPs inhibited the pathogen’s cell viability, shown by the absence of the reduction process of AB dye after 24 h. The pathogen growth was inhibited by 76.8% and 77.7% at Cu-NP concentrations of 200 and 500 mg/mL, respectively (Figure 2B). With the exception of Cu-NPs, all the other used forms of copper did not affect the pathogen’s viability. Interestingly, when the incubation time was extended to 48 h, the *AB* dye in the group treated with Cu-NPs (200 mg/mL) turned gradually to purple, but remained unchanged at 500 mg/mL, suggesting that Cu-NPs at 200 mg/mL may be insufficient to inhibit the viability of the fungus.

### 3.3. X-ray Diffraction (XRD) Analysis of Copper forms

**Figure 3:**
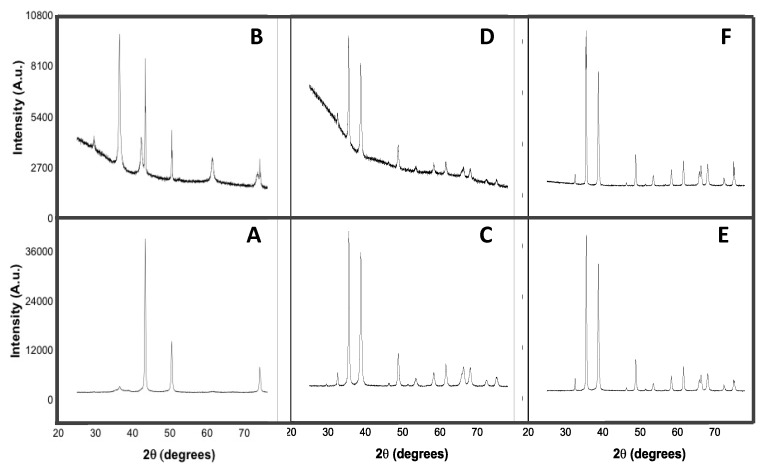
X-ray diffraction (XRD) patterns of Cu-NPs, CuO-NPs, and CuO before (respectively **A**,**C**,**E**) and after (**B**,**D**,**F**, respectively) interaction with the *C. gloeosp* for seven days. Patterns were obtained with a PANalytical X’PERT PRO MPD diffractometer (Malvern Panalytical, Japan) operating at 45 kV and 40 mA with Cu-Kα radiation (λ = 1.54060 Å) at a scan speed of 0.01 from the 2θ 20° to 80° range.

X-ray diffraction patterns of Cu-NPs, CuO-NPs, and CuO were obtained to examine the initial physical characteristics of the metals (Figure 3). Analysis was also conducted after interaction with the fungus in order to learn of any changes that may have occurred after interaction with the fungus. 

As can be observed from the pattern, three major diffraction peaks were obtained for Cu-NPs at 2θ 43.29°, 50.41°, and 74.09°. They were indexed to (111), (200), and (222) planes, respectively, which were in a good agreement with a face centered cubic structure of Cu-NPs with traces of Cu_2_O (International Centre of Diffraction Data (ICDD) card no. 74-5761). After interaction with the pathogen, eight diffraction peaks were observed. The major reflections at 2θ 43.31°, 50.45°, and 74.13° could be assigned to the (111), (200), and (220) planes, respectively, similar to the characteristics of Cu-NPs before interaction with the fungus. The remaining peaks at 2θ 29.55°, 36.99°, 42.27°, 61.33°, and 73.46° corresponded to the (110), (111), (200), (220), and (311) crystal of planes Cu_2_O (ICDD no. 77-7719).

Before use, the CuO-NP XRD spectrum presented 12 diffraction peaks at 2θ 32.53°, 35.61°, 38.77°, 46.28°, 48.80°, 53.48°, 58.29°, 61.57°, 66.14°, 68.04°, 72.43°, and 75.12°. They were indexed to the (110), (−111), (111), (−112), (−202), (020), (202), (−113), (−311), (220), (311), and (−222) planes, and matched to the monoclinic phase of CuO with space group C2/c without impurities (ICDD no. 70-6831). In comparison with the initial diffractogram, after interaction with the fungus, all reflection peaks and their indexed planes were unchanged, with the exception at 2θ 46.28° and 72.43°. However, the physical features of the compound remained the same (ICDD no. 45-0937).

The XRD patterns obtained for CuO before and after contact with the fungus matched ICDD no. 45-0937 and no. 89-5895, respectively. Both patterns corresponded to a monoclinic phase of tenorite CuO with space group C2/c. Originally, 14 reflection peaks at 2θ 32.54, 35.53, 38.77°, 46.28°, 48.79°, 51.41°, 53.49°, 58.33°, 61.57°, 65.81°, 66.30°, 68.04°, 72.44°, and 75.09° were obtained and indexed to the (−110), (002), (111), (−112), (−202), (112), (020), (202), (−113), (022), (−311), (−220), (311), and (004) crystal planes, respectively. After interaction with the pathogen, all reflection peaks remained unchanged, except for the one at 2θ 51.41°.

### 3.4. Effects of Copper Forms on the Ultrastructure of the Fungus

**Figure 4:**
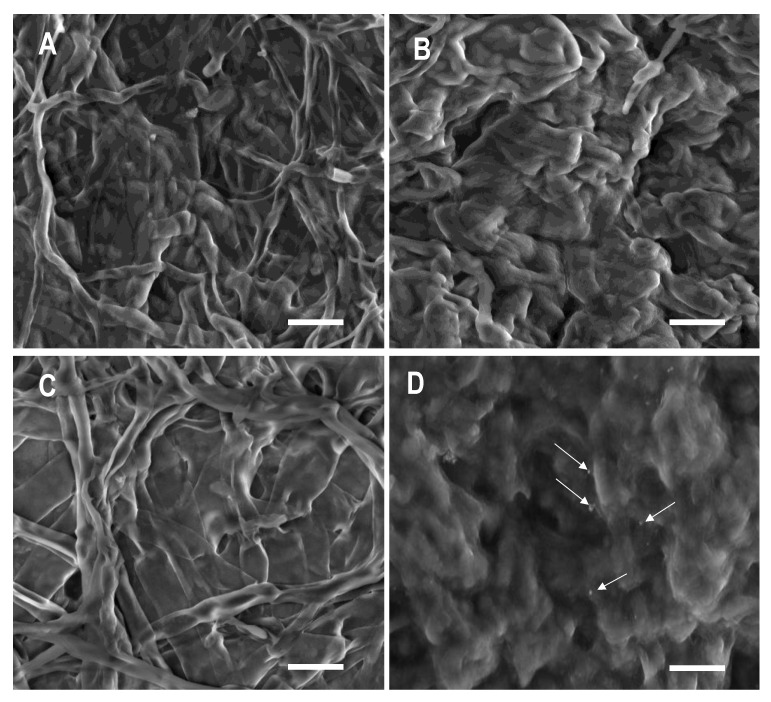
Scanning electron micrographs of *C. gloeosp* mycelium (**A**) No treatment (control), (**B**–**D**) Cu-NP, CuO, and CuO-NP treatments, respectively, at 500 mg/mL. In (**D**), arrows point to small NPs present on the surface of the pathogen’s vegetative part. Pictures were obtained using a FEI Quanta 400 (Thermo Fisher Scientific, Japan) operating in low vacuum with water vapor and an accelerating voltage of 10 kV at 4000x magnification. Scale bar = 40 μm.

Finally, the ultrastructure of the pathogen’s hyphae before and after exposure to the metals was examined in an attempt to understand the control mechanism of the copper forms (Figure 4). From the obtained micrographs, the disruption of cell integrity, especially the vegetative parts, could be clearly observed. It is difficult to rate the degree of cell disruption as it relates to functionality. Thus, the observed physical changes can only be described in relation to the control. Later in the discussion section, an attempt to relate these physical changes to the effects of the copper compounds on fungal growth and viability will be made.

*C. gloeosp*. exhibits a well-defined branched tubular hyphae. In the presence of all forms of copper, the hyphae had a bigger and fuller/swollen appearance. In the presence of CuO, although the typical branched hyphal structure could still be observed, it was swollen and had lost its well-defined tubular shape. When treated with the nanoforms of copper, most of the hyphae seemed to have fused to a molten larvae appearance, especially with CuO-NPs where no discernable hyphae could be observed. In addition, small nanoparticles were present on the surface of the pathogen’s vegetative part. Among all treatments, CuO appeared to have the least effect on the pathogen.

## 4. Discussion

In agricultural sciences, metallic nanomaterials have started to play a significant role in crop protection because of their unique physical and chemical properties including a huge surface to volume ratio, structural stability, and strong affinity to their target [49]. Among the leading metallic nanomaterials, copper is the most popular and so far, the only solid antimicrobial material registered. Copper is biocompatible, and is an important element in some physiological processes. As such, it holds great potential for use alone, or for integration into existing fungicides and control measures, for enhanced and with eco- and bio-sensitive performance. Incorporation of copper and CuO in fungicide for treatment against *C. gloeosp* have also been studied, with effects shown on mycelial growth [50]. Rampersad and Teelucksingh reported that increasing concentrations of copper fungicide inhibited spore survival for *Colletotrichum* isolates [34].

Numerous studies have reported the antimicrobial activity of Cu-NPs against several fungi [43,44,45,46]. The results of the present study indicate that all of the copper forms inhibited hyphal growth in a dose dependent manner, with Cu-NPs and CuO-NPs showing the most inhibiting effect against the pathogen. Reports on the effect of copper-based treatments on fungi, especially *C. gloeosp,* are very few and relatively new, indicating that the use of this metal is highly topical, novel, and interesting. The recent reports have shown Cu-NPs to be effective in inhibiting the growth and colony formation of *C. gloeosp,* in agreement with our work [42,45]. Furthermore, similar to our findings, Malandrakis et al. reported that the oxidative forms of Cu-NPs were less effective than Cu-NPs [45]. The studies have been limited to the effects of copper forms on the growth of the fungus and inhibition of colony formation. In addition to studying the effects of nanostructured copper (CuO-NPs, Cu-NPs) and CuO on the vegetative growth of the pathogen, we also investigated the effects on cell viability for the first time. The Alamar Blue assay has been of primordial importance in determining the efficacy of the metal particles against the viability of fungus cells. Our findings revealed that only Cu-NPs were effective in inhibiting *C*. *gloeosp.* cell viability. CuO-NPs and its bulk form (CuO) did not inhibit the pathogen’s cell proliferation, strongly suggesting the long-term effects (and sustainability) of Cu-NPs for the management of *C. gloeosp.* This suggests potential differences in the mode of action of the oxidized compared to normal forms of nanoparticle materials. Limited and still controversial information exists regarding the antimicrobial activity of CuO-NPs. 

The underlying mechanism by which copper materials kill fungi is not fully clear, but the toxicity of copper particles depends on the combination of several factors such as concentration, length of exposure, humidity, and temperature [6,26]. It has been reported that copper interacts with microorganisms in various ways including cell membrane permeabilization, membrane lipid peroxidation, protein alteration, and denaturation of nucleic acids, ultimately leading to cell death. Weaver et al. [8] and Grass et al. [35] demonstrated that copper provided an antifungal surface and a contact killing mechanism was observed, with virtually no live microorganisms recovering from the copper surfaces after prolonged exposure. It was proposed that the contact-mediated killing starts with extensive fungal membrane damage, followed by cell vacuole enlargement and disappearances [51]. Going forward, we are conducting experiments using model membrane systems to help elucidate the mechanism of membrane breach and/or damage including changes in molecular re-organization and fluidity. Copper NP and AgNP aggregation/dissolution have been reported to occur under physiological conditions [52,53]. Thus, we will also investigate the possibility of copper aggregation/agglomeration in media and upon interaction with the fungus in order to understand any effect that particle size change may have on the antifungal activity of copper. This may also help in elucidating the antifungal mechanisms involved. 

XRD studies on the crystal structure of copper forms have shown changes in the chemo-physical features of Cu-NPs (Figure 3). Sixty percent of Cu-NPs were oxidized into Cu_2_O during the interaction with the pathogen. Diffractograms clearly showed that only Cu-NPs had undergone extensive chemical transformation in comparison with CuO-NPs and CuO. Interestingly, these observations are closely correlated to the cell viability assay outcomes described earlier. They may hold clues into the differences of the mode of action between nano-sized copper (Cu-NPs) and their oxidized forms (CuO-NPs and CuO). Since the difference in the sizes of the copper nanoforms were less than an order of magnitude, we propose that the oxidation process could be important toward how Cu-NPs affect *C. gloeosp*’s growth as well as the viability of the cells. Finally, we imaged the morphological transformation of the fungus before and after interaction with each of the copper forms. Micrographs of mycelia showed that all treatments had an effect on mycelia, with groups treated with the nanoforms of the metal (Cu-NPs and CuO-NPs), showing extensive deformation compared to those treated with the bulk metal (CuO). These observations are consistent with the hyphal growth inhibition pattern observed and discussed earlier. Interestingly, CuO-NP appeared to cause slightly more mycelial deformation than Cu-NPs. Our follow-up research using model membrane systems, Fourier Transform InfraRed spectroscopy (FTIR) analyses, and further characterization of the nanostructured copper forms such as particle size distribution and zeta potential may help elucidate mechanisms involved for each of the copper forms, and aid in understanding how the fungal mycrographs relate to cell viability and hyphal growth as well as the effect of Cu-NP oxidation. 

Overall, the present study demonstrated that Cu-NPs, CuO-NPs, and CuO possess antifungal ability against *C. gloeosp*. with the nanoforms having a high inhibiting effect on the pathogen hyphal growth. In contrast to its oxidized form, only Cu-NPs have potent ability to prevent *C. gloeosp.* spore germination. To be the best of our knowledge, this is the first study to report (i) the differences in the effect of Cu-NPs, CuO-NPs, and CuO against *C. gloeosp*.; (ii) the effectiveness of Cu-NPs for controlling the fungus, as it stops the cells viability, and (iii) the formation of an oxidative form of copper after Cu-NP interaction with fungus. The obtained results may open opportunities for the application of biocompatible metal NPs for the control and management of economically-devasting anthracnose diseases.

## Figures and Tables

**Table 1:** Effects of copper forms, copper concentrations, and the interactions between both factors on the fungal hyphal growth (Analysis of Variance test).

Variables	df	F	Significance
Type of copper	2	38.7	<0.05
Concentration of copper	4	401.1	<0.05
Type of copper and copper concentration	8	8.2	<0.05

## Supplementary Materials

The following are available online at https://www.mdpi.com/2079-4991/10/5/1003/s1, They include optimisation of Alamar Blue assay, Table S1: Effects of spore density, medium and incubation period on percent reduction of Alamar Blue dye (Analysis of variance, NOVA test), Figure S1: Effects of spore density and type of media on percent reduction of Alamar Blue dye, Figure S2: Effects of spore density, medium and incubation period on visual color change of Alamar Blue dye.
