# Supplementary material for: Antifungal Potential of Nanostructured Crystalline Copper and Its Oxide Forms
Source: Nanomaterials (Basel). 2020 May 24;10(5):1003. doi: 10.3390/nano10051003 (PMC7279545; doi:10.3390/nano10051003)
Supplement: Supplementary file 1 [file nanomaterials-10-01003-s001.pdf]

Supplementary Materials for

Antifungal Potential of Nanostructured Crystalline Copper and Its Oxide Forms

Auriane Fifame Oussou-Azo [1], Tomoki Nakama [2], Masayuki Nakamura [1,2], Taiki Futagami [1,2] and Mun'delanji Catherine M. Vestergaard [1,2,]*

[1] United Graduate School of Agricultural Sciences, Kagoshima University, Kagoshima 890-0065, Japan; oauriane1@gmail.com (A.F.O.-A.); masa@agri.kagoshima-u.ac.jp (M.N.); futagami@chem.agri.kagoshima-u.ac.jp (T.F.)

[2] Faculty of Agriculture, Kagoshima University, 1-21-24 Korimoto, Kagoshima 890-0065, Japan; nt11uver@gmail.com (T.N.)

* Correspondence: munde@agri.kagoshima-u.ac.jp

Alamar Blue (*AB*) assay optimization: Five densities of pathogen conidia suspension (10^1 to 10^5 spores/mL) were incubated at four different periods of time (6, 12, 18 and 24 h) using potato dextrose broth (PDB) and 10 % (v/v) V8 juice broth. The type of medium, the spore density and the incubation time, all significantly ($P < 0.05$) affected the percent reduction of *AB*, and consequently, the pathogen's proliferation (Table S1). Regardless of incubation time, 10 % (v/v) V8 broth ensured a better proliferation of the pathogen than PDB at all spore densities (Figure S1). However, A*B* reduction in PDB had a steadier gradient, increasing from -20 to 60 along with the cell density. In the case of, 10 % V8 broth, reduction of *AB* only increased from 60 to 80, with decreases seen between 10^1-10^2 spores/mL and 10^3-10^4 spores/mL. These observations are in agreement with the visual inspection of the plates (Figure S2), where the dye turned from blue to pink only after 6 h of sample incubation and to light pink at 18 h. This may explain why there was a quick start of a second redox phase of *AB* in 10 % V8 broth. Based on these results, PDB was selected for *AB* assay. In PDB, the effective color change of AB occurred after 18 h of incubation, but the highest gradient was observed after 24 h ($R^2 = 0.735$ vs $R^2 = 0.435$ at 18 h) with the calibration curve being linear between 10^2 to 10^5 spores/ mL. Consequently, we chose 24 h of incubation time and 10^5 spore density for the AB assay experiments on the effect of the various copper forms with on the target pathogen.

Table S1. Effects of spore density, medium and incubation period on percent reduction of Alamar Blue dye (Analysis of variance, NOVA test)

Variables	df	F	Significance
Incubation period	3	8.4	< 0.05
Type of Media	1	35.5	< 0.05
Spore Density	4	10.2	< 0.05

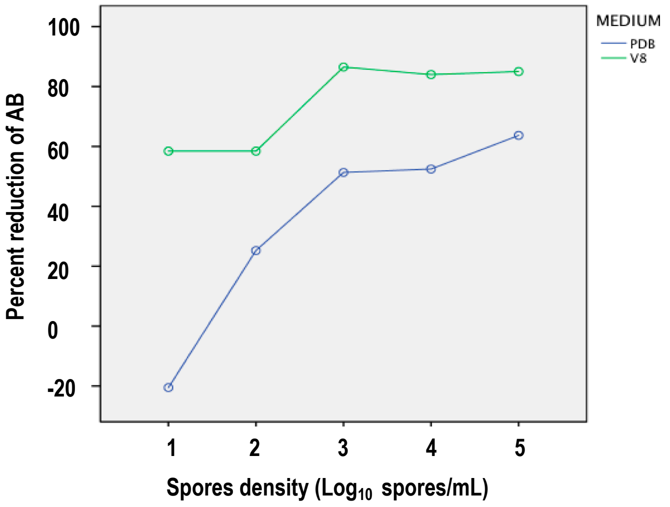

Figure S1. Effects of spore density and type of media on percent reduction of Alamar Blue dye.

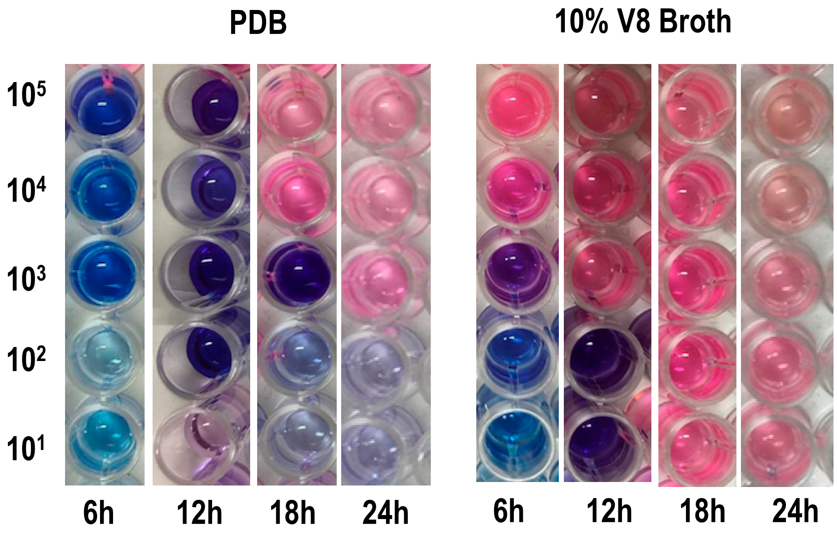

Figure S2. Effects of spore density, medium and incubation period on visual color change of Alamar Blue dye.
